# Prioritization of a plant polysaccharide over a mucus carbohydrate is enforced by a Bacteroides hybrid two-component system

**DOI:** 10.1111/j.1365-2958.2012.08123.x

**Published:** 2012-07-05

**Authors:** Jonathan B Lynch, Justin L Sonnenburg

**Affiliations:** Department of Microbiology and Immunology, Stanford University School of MedicineStanford, CA 94305, USA.

## Abstract

Bacteroides is a dominant genus within the intestinal microbiota of healthy humans. Key adaptations of the Bacteroides to the dynamic intestinal ecosystem include a diverse repertoire of genes involved in sensing and processing numerous diet- and host-derived polysaccharides. One such adaptation is the carbohydrate-sensing hybrid two-component system (HTCS) family of signalling sensors, which has been widely expanded within the Bacteroides. Using *Bacteroides thetaiotaomicron* as a model, we have created a chimeric HTCS consisting of the well-characterized sensing domain of one HTCS, BT1754, and the regulatory domain of another HTCS, BT0366, to explore the regulatory capabilities of these molecules. We found that the BT0366 regulatory region directly binds to and mediates induction of the adjacent polysaccharide utilization locus (PUL) using whole-genome transcriptional profiling after inducing signalling through our chimeric protein. We also found that BT0366 activation simultaneously leads to repression of distal PULs involved in mucus carbohydrate consumption. These results suggest a novel mechanism by which an HTCS enforces a nutrient hierarchy within the Bacteroides via induction and repression of multiple PULs. Thus, hybrid two-component systems provide a mechanism for prioritizing consumption of carbohydrates through simultaneous binding and regulation of multiple polysaccharide utilization loci.

## Introduction

The human intestine is home to a dense community of microbes, together known as the intestinal microbiota. Hundreds of bacterial species reside side-by-side at densities exceeding 10^11^ cells ml^−1^, creating a complex and competitive environment (Backhed *et al*., 2005). This microbial ecosystem is subject to constant disruption due to many factors, including changes in host diet. The perfusion of the intestine with continually changing dietary nutrients results in a dynamic ecosystem to which the residents must constantly adjust. The domination of the healthy human microbiota by the Bacteroidetes and the Firmicutes suggests that the resident members of these two phyla are especially well adapted to respond to the challenges posed within the intestinal environment (Eckburg *et al*., 2005; Ley *et al*., 2008).

*Bacteroides thetaiotaomicron* (*Bt*) is a prototypic member of the *Bacteroides* genus within the Bacteroidetes phylum and is an abundant species within the human intestinal microbiota (Eckburg *et al*., 2005; Qin *et al*., 2010). *Bt* possesses several genomic features characteristic of the genus, such as expanded repertoires of polysaccharide utilization loci (PULs) (88), glycoside hydrolases (> 260) and PUL-associated signalling systems (> 60), including 32 members of the hybrid two-component system (HTCS) family (Xu *et al*., 2003; 2007). Accordingly, *Bt* has displayed the ability to process a wide range of polysaccharides present in the plant components of the human diet, as well as mucosal glycans that are exposed on the apical surface of the host intestine and secreted into the lumen (Salyers *et al*., 1977; Sonnenburg *et al*., 2005; Martens *et al*., 2008). Whole-genome expression profiling of *Bt* at multiple time points during growth in rich media revealed that induced PULs are sequentially upregulated at specific times throughout growth, rather than concurrently (Sonnenburg *et al*., 2006). In several instances, PUL-associated signalling systems have been implicated in sensing glycans and directing upregulation of their neighbouring PULs, but the mediators of this hierarchical PUL expression and prioritization of polysaccharide use remain poorly understood (Martens *et al*., 2008; Sonnenburg *et al*., 2010).

**Fig. 1:**
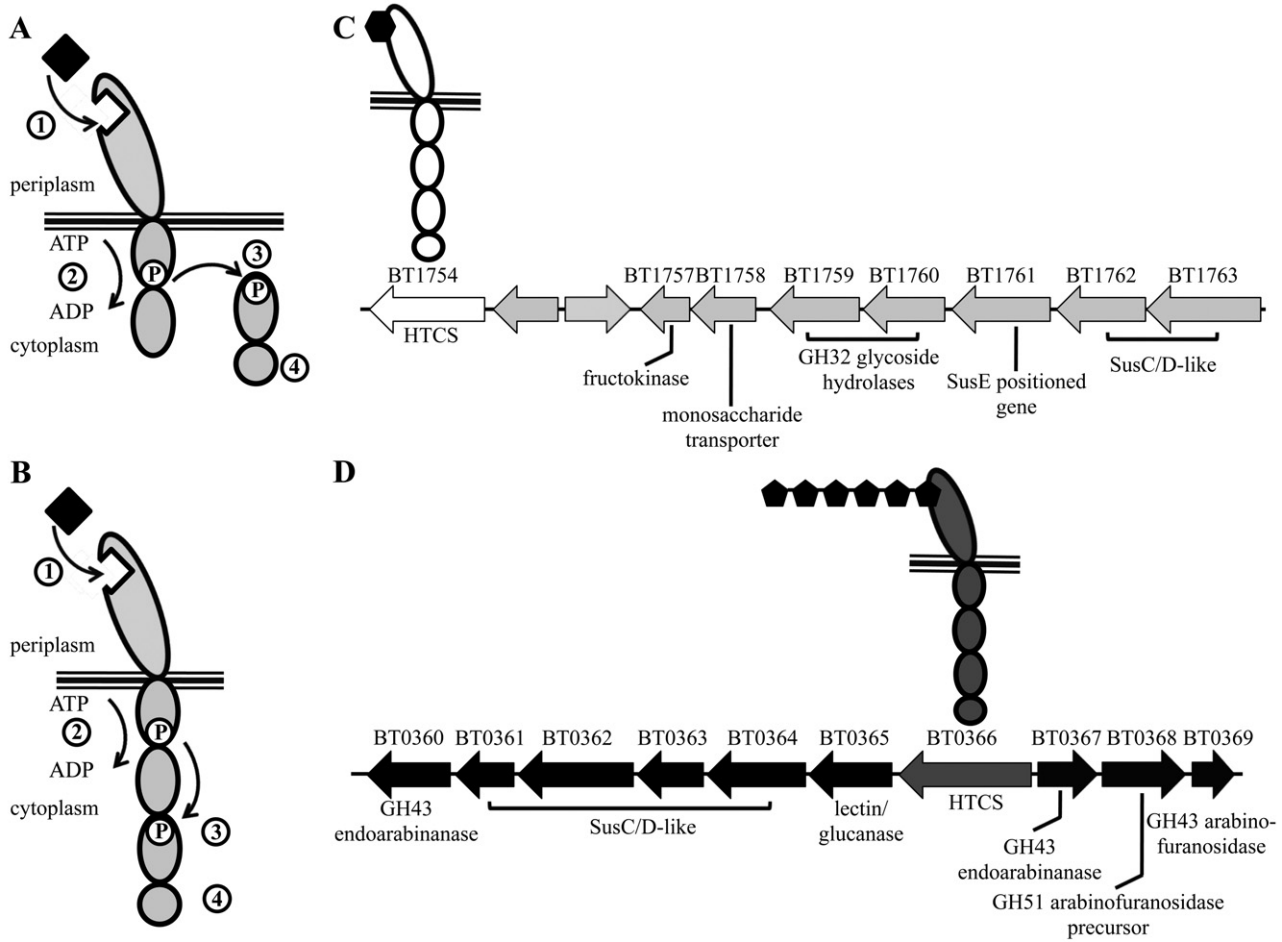
Schematic of the hybrid two-component system (HTCS) family of signalling sensors. A. Schematic of canonical two-component system. An environmental signal, such as a nutrient ligand, binds to the extracellular sensor domain (1), resulting in intracellular phosphorylation of a histidine residue on the phosphoacceptor domain (2). The phosphate is then transferred to an aspartic acid on the response regulator (3). The resulting activated response regulator affects cellular activity via an output domain (4), often by promoting transcription. B. Schematic of an HTCS. HTCSs contain all of the domains typically found in the two proteins of a canonical two-component system encoded by a single gene. Additionally, they characteristically contain an output domain that consists of a helix–turn–helix DNA binding module of the AraC family (4). C. Schematic of BT1754, a well-described HTCS in *Bt*, and its associated PUL (adapted from Sonnenburg *et al*., 2010). The periplasmic sensing domain binds monomeric fructose. BT1754 is required for the upregulation of the adjacent fructan utilization PUL and for efficient use of fructose-based carbohydrates. D. Schematic of BT0366, another HTCS in *Bt*, and its adjacent PUL. We show BT0366 binding to a linear arabinan-derived hexameric oligosaccharide, one of several arabinan-derived ligands it has been shown to bind (Martens *et al*., 2011). The *BT0366* gene sits within a PUL consisting of two divergently encoded gene cassettes with several genes shown to be involved in processing various forms of arabinan (Cartmell *et al*., 2011).

Two-component systems are a common means by which bacteria sense and respond to environmental stimuli. Ligand binding to the periplasmic sensor domain initiates activation of the intracellular histidine kinase domain (pFam category HATPase_c), which in turn leads to phosphorylation of a histidine residue in a phosphoacceptor domain (HisKA). This phosphate is subsequently transferred to an aspartic acid on a second protein known as a response regulator, presumably leading to an activating conformational change that enables the response regulator to impact some cellular event, often by behaving as a transcription factor for a stimulus-specific response (Fig. 1A) (Stock *et al*., 2000). These multi-protein signal relays have been found to be involved in processes such as symbiosis, virulence, biofilm formation, competence, cell division and antimicrobial production (Domian *et al*., 1997; Belanger *et al*., 2009; Martinez-Nunez *et al*., 2010; Petrova and Sauer, 2010; Senadheera *et al*., 2012). Two-component systems are often part of complex regulatory networks that involve interactions between many different regulatory systems, providing a complex and dynamic responsive capacity beyond that of individual sensing systems (Biondi *et al*., 2006).

While canonical two-component systems have been extensively studied, much HTCS biology, including the scope of their respective regulatory networks, remains unknown despite their apparent importance in the physiology of many intestine-adapted symbionts. HTCSs combine the functional units of canonical two-component systems into one protein that is theoretically capable of signal perception, phosphorelay, and ultimately mediating transcriptional events through an HTH_AraC DNA-binding domain (Fig. 1B) (Xu *et al*., 2004; Sonnenburg *et al*., 2006). Due to the fact that HTCSs are each encoded by a single gene, they likely have a set of regulatory attributes distinct from those of canonical two-component systems. Previously, we have shown that BT1754, the HTCS associated with *Bt*'s fructan utilization PUL, binds to fructose and is required for fructose-induced upregulation of the adjacent PUL (Sonnenburg *et al*., 2010). Structural and biochemical studies of BT1754 revealed that the periplasmic sensor domain directly binds monomeric fructose. Despite the documented requirement of *BT1754* for the adjacent fructan PUL upregulation, it has been difficult to address whether it and other HTCSs mediate other regulatory events throughout the genome. A primary challenge is distinguishing the HTCS-dependent regulatory events from those that are merely coincident with HTCS activation such as the presence and catabolism of the activating carbohydrate. In addition, deletion of *BT1754* results in the inability of *Bt* to grow in fructose, which limits the insight gained from gene deletion studies.

Including BT1754, 28 of *Bt*'s 32 HTCS genes are located adjacent to PULs within the *Bt* genome, suggesting they play a widespread role in regulating these gene sets (Martens *et al*., 2008). In contrast to the well-characterized fructose-binding HTCS BT1754 (Fig. 1C), the HTCS *BT0366* and its adjacent locus have only recently been studied in detail (Fig. 1D). The *BT0366*-associated locus contains the genes characteristic of a PUL, including homologues of *susC* and *susD* and several glycoside hydrolases shown to act on arabinan derivatives (glycoside hydrolase families 43 and 51) (Cartmell *et al*., 2011). Recent work has established a carbohydrate ligand for BT0366 signalling (Martens *et al*., 2011), but the global regulatory impact of BT0366 activation has not been previously examined.

Here, we use a chimeric HTCS in which we have fused the well-defined periplasmic sensor domain of BT1754 with the uncharacterized intracellular regulatory region of BT0366. In *E. coli*, similar chimeric receptors have been used to create various combinations of the canonical two-component system sensors Tar, NarX, Trg and EnvZ to explore sensor structural conservation, the effects of phosphorylation/dephosphorylation activity on downstream signalling, and ligand specificity (Utsumi *et al*., 1989; Yang and Inouye, 1993; Baumgartner *et al*., 1994; Ward *et al*., 2002; Michalodimitrakis *et al*., 2005). Our chimera utilizes a similar strategy of fusing domains from the two proteins at a conserved region of the predicted cytoplasmic linker domains, presumably avoiding disruption of the domains most important for normal ligand binding, phosphate transfer and signal relay. Importantly, the chimeric HTCS allows us to uncouple carbohydrate signalling (induced by fructose) from carbohydrate utilization, which enables the regulatory network of BT0366 to be defined under controlled conditions. We use the chimeric HTCS to demonstrate that carbohydrate-driven HTCS activation results in upregulation of the *BT0366*-adjacent PUL, which is involved in the degradation and import of the dietary plant glycan arabinan. In addition, we use whole-genome expression profiling and chromatin immunoprecipitation to discover that the activated chimeric HTCS simultaneously binds to and represses distal PULs involved in host glycan utilization. Thus, we propose a model in which HTCSs can enforce a carbohydrate utilization hierarchy via two distinct activities. Here, HTCS activation results in (i) upregulation of the adjacent PUL to enable carbohydrate consumption, and (ii) the concurrent repression of PULs associated with the use of lower priority carbohydrates.

## Results

### Arabinan-dependent upregulation of the PUL BT0360–BT0369 requires the associated HTCS BT0366

We set out to identify a commercially available carbohydrate substrate to which the BT0366-associated PUL (*BT0360–0369*) is transcriptionally responsive. *Bt* cultures grown in minimal media (MM) supplemented with purified carbohydrates as the sole carbon and energy source were harvested at mid-log phase. Quantitative RT-PCR was performed on cDNA generated from each sample to determine the PUL expression level. We tested several arabinose-based substrates including arabinose (a monosaccharide), arabinogalactan (a plant polysaccharide comprised of a β1–3 galactose backbone with branches of mostly β1–6 galactose and β1–3 arabinose), and arabinan (a plant polysaccharide composed of α1–5 arabinofuranose with a high number of α1–2 and α1–3 arabinofuranose substitutions). As controls, we also grew *Bt* in MM-glucose or MM-fructose and surveyed expression of the fructose-responsive fructan utilization locus associated with *BT1754*.

**Fig. 2:**
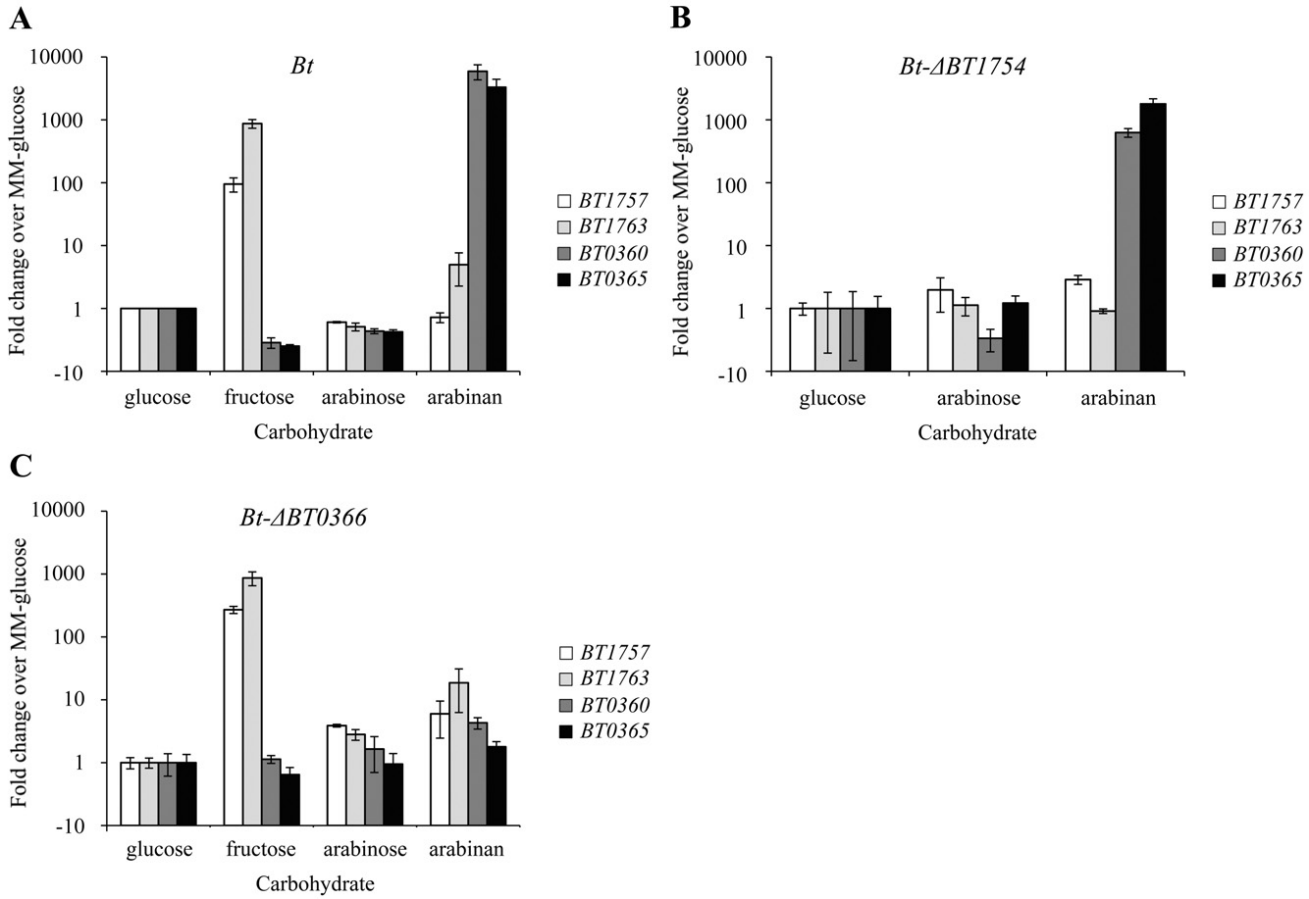
Arabinan induces the *BT0366*-associated PUL in a *BT0366*-dependent manner. Relative expression of genes from the *BT1754*-associated PUL (*BT1757*, *BT1763*) and the *BT0366*-associated PUL (*BT0360*, *BT0365*) in *Bt* (A), *Bt-*Δ*BT1754* (B) and *Bt-*Δ*BT0366* (C) as determined by qRT-PCR. Values displayed are fold change relative to that strain grown in MM-glucose. Error bars are ± SE. MM-fructose was omitted for *Bt-*Δ*BT1754* due to the strain's growth defect in this condition.

Consistent with recent work (Martens *et al*., 2011), the locus associated with *BT0366* is upregulated during growth in arabinan (*BT0360*: 5914.3-fold induction, *P* = 0.006; *BT0365*: 4426.8-fold induction, *P* = 0.01), but is not affected by fructose, arabinose or arabinogalactan (Fig. 2A and data not shown). These results confirm that the *BT0366*-adjacent PUL responds to the polysaccharide arabinan, but unlike the fructan PUL, which is transcriptionally responsive to a monosaccharide (fructose) derived from the substrate it degrades, the *BT0366*-associated PUL is not responsive to the monosaccharide product of arabinan hydrolysis, arabinose. Our results also confirmed that the fructan utilization PUL adjacent to *BT1754* is significantly upregulated in the presence of fructose (*BT1757*: 95.1-fold induction, *P* = 0.003; *BT1763*: 707.4-fold induction, *P* = 0.004) when compared with growth in glucose, as we have previously shown (Fig. 2A) (Sonnenburg *et al*., 2010). Arabinose, arabinogalactan (data not shown) and arabinan have no significant effects on this fructan utilization locus (Fig. 2A).

In order to determine if *BT0366* is required for upregulation of its neighbouring PUL, we created a strain of *Bt* in which *BT0366* is genetically deleted (*Bt*-Δ*BT0366*; see *Experimental procedures*). We surveyed gene expression of the *BT0366*-associated PUL by qRT-PCR in *Bt* and *Bt*-Δ*BT0366* grown in MM-arabinan compared with MM-glucose. The upregulation of the arabinan-associated PUL is lost upon *BT0366* deletion, demonstrating that *BT0366* is required for upregulation of its neighbouring PUL in response to arabinan (Fig. 2C). The *Bt*-Δ*BT0366* strain exhibits significantly impaired growth in arabinan alone, while maintaining normal growth in other carbon sources, consistent with a recent report (Martens *et al*., 2011) (Fig. S1). Deletion of *BT1754* from *Bt* strongly delays its growth in fructose, but has no effect on the arabinan-dependent growth (Fig. S1) or induction of the *BT0366*-associated PUL (Fig. 2B). The fact that *Bt-*Δ*BT1754* and *Bt-*Δ*BT0366* strains are each specifically impaired in the upregulation of their respective PULs illustrates the specificity and independence of HTCS sensing and response.

### Fructose and BT1754 mediate repression of BT0366-associated PUL

**Fig. 3:**
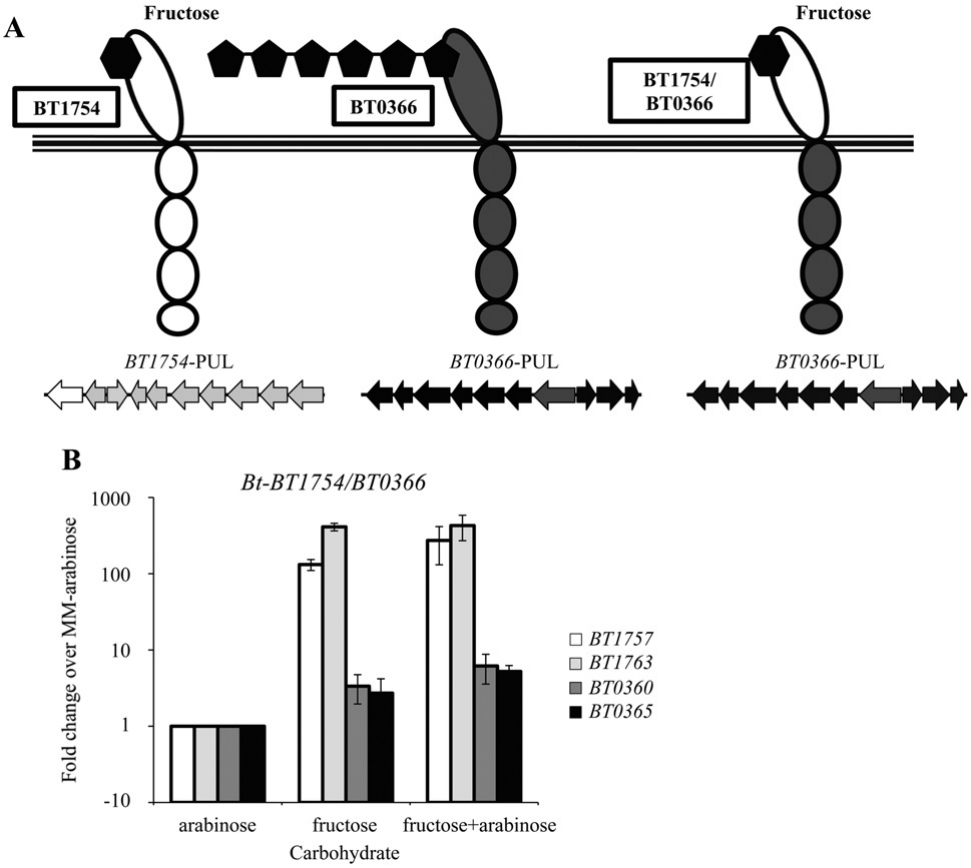
The *BT1754/BT0366* chimeric HTCS is not sufficient to activate the *BT0366*-associated PUL in *Bt* in a fructose-dependent manner. A. Schematic of the BT1754/BT0366 chimera, where the periplasmic fructose-sensing domain of BT1754 was fused to the intracellular phosphotransfer and response regulator domains of BT0366. B. Relative expression levels of genes from the *BT1754*-associated PUL (*BT1757*, *BT1763*) and the *BT0366*-associated PUL (*BT0360*, *BT0365*) from the chimera-expressing strain *Bt-BT1754/BT0366* grown in MM supplemented with fructose, arabinose, or both, as assessed by qRT-PCR. Values displayed are mean fold changes in expression compared with *Bt-BT1754/BT0366* grown in MM-arabinose. Error bars are ± SE.

We created a chimeric HTCS containing the well-characterized periplasmic fructose-sensing domain of BT1754 (aa 1–376; includes TM and first 14 predicted cytoplasmic residues) and the intracellular phosphotransfer and regulatory region of BT0366 (aa 855–1420) to investigate HTCS signalling (Fig. 3A). This splice junction was chosen by sequence alignment and basic structure predictions to maintain protein domain integrity and proper membrane localization (another chimera consisting of aa 1–808 of BT1754 and aa 1302–1420 of BT0366 did not successfully signal and was not considered further for these studies). The BT1754/BT0366 chimera allowed us to circumvent two problems associated with the investigation of HTCS-mediated regulation. First, the chimeric HTCS allowed us to directly assay the influence of the BT0366 regulatory region using the previously characterized ligand to BT1754, fructose. Second, by uncoupling processing of the HTCS ligand from the activities that are upregulated by HTCS activation, we could more easily separate confounding BT0366-independent events, such as catabolite repression or signalling from other sensors, from responses directly mediated by BT0366 activation. Specifically, we would not expect arabinan-use genes to be upregulated during growth in fructose unless they are induced by the BT1754/BT0366 chimera. Thus, we can infer any responses that depend upon both the BT1754/BT0366 chimera and fructose are likely mediated by the activated regulatory domains of BT0366.

We first wished to test whether fructose could induce expression of the *BT0366*-associated PUL in the strain harbouring the chimeric HTCS (*Bt-BT1754/BT0366*). The chimeric HTCS construct was designed to include the intergenic region at the 5′ end of the native *BT1754* to drive expression of the chimera (see *Experimental procedures*). The *BT1754* promoter embedded within this region is not responsive to fructose, but rather results in low levels of constitutive expression (Sonnenburg *et al*., 2010). The *Bt*-*BT1754/BT0366* strain was grown in fructose and expression of genes within the *BT1754*- and *BT0366*-associated PULs were assayed by qRT-PCR. While the *BT1754*-associated PUL was upregulated as expected, we saw little induction of the *BT0366*-associated PUL in *Bt-BT1754/BT0366*, suggesting that fructose was not sufficient to induce chimera signalling in these conditions (Fig. 3B).

Carbohydrate prioritization via repression of operons that are involved in the use of lower priority substrates is common in bacteria. Previously, whole-genome expression profiling has been used to illustrate the ability of *Bt* to prioritize several different types of carbohydrates over the course of growth in rich medium (Sonnenburg *et al*., 2006). However, the mechanisms involved in co-ordinating this sequential upregulation of multiple PULs have not been elucidated. Therefore, we reasoned that one possible explanation for the inability of our chimera to upregulate the *BT0366*-associated PUL is that endogenous use of fructose by *Bt* mediates repression of these genes.

**Fig. 4:**
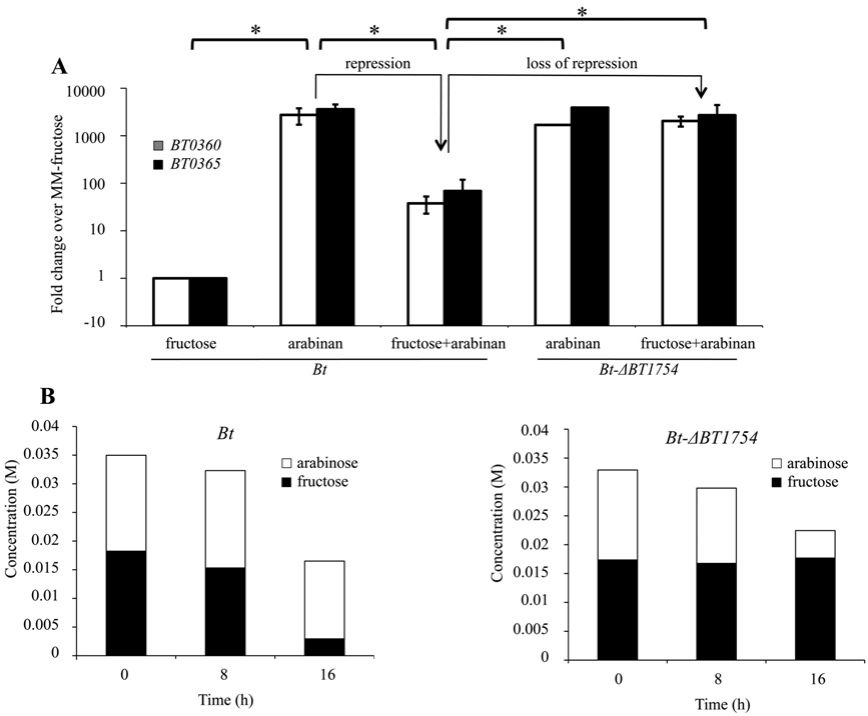
Repression of the *BT0366*-associated PUL depends on fructose and *BT1754*. A. Relative expression levels of genes from the *BT0366*-associated PUL (*BT0360*, *BT0365*) in *Bt* or *Bt-*Δ*BT1754* strains grown in MM-fructose, MM-arabinan or MM-fructose + arabinan, as determined by qRT-PCR. Values displayed are mean fold changes in expression relative to *Bt* grown in MM-fructose. Error bars are ± SE. *P < 0.05. The repression of the *BT0366*-associated PUL that depends upon fructose and *BT1754* and its loss upon deletion of *BT1754* is noted with arrows. B. Monosaccharide content of media at various time points after inoculation with *Bt* (left panel) or *Bt*-Δ*BT1754* (right panel) grown in MM-fructose + arabinose, as determined by HPAEC-PAD. Values represent concentration of the indicated sugar remaining in supernatants collected from growths at specified time points in molar (M).

We next tested whether the presence of fructose in the medium was leading to repression of *BT0366*-associated genes. *Bt* was grown in minimal medium supplemented with 0.25% fructose plus 0.25% arabinan, known inducers of the *BT1754*-associated PUL and the *BT0366*-associated PUL respectively. In the presence of both fructose and arabinan, the *BT1754*-associated genes were induced as expected (Fig. S2). However, in the mixture of the two carbohydrates, *BT0366*-associated genes exhibited significantly reduced expression compared with expression levels observed when *Bt* was grown in arabinan alone, consistent with fructose-mediated repression of the *BT0366*-associated PUL (Fig. 4A). To test if *Bt* prioritizes fructose over arabinose (the product of arabinan hydrolysis) when both are present in the environment, we surveyed depletion of the carbohydrates during growth. High pH Anion Exchange Chromatography with Pulsed Amperometric Detection (HPAEC-PAD) was used to monitor the concentration of fructose and arabinose remaining in the medium during growth of *Bt*. These data revealed that *Bt* depleted fructose more rapidly than arabinose, consistent with carbohydrate prioritization of fructose over arabinose when both are present in the medium (Fig. 4B, left panel).

We next deleted *BT1754* to determine if elimination of this HTCS would alleviate repression of the *BT0366*-associated genes during growth in fructose plus arabinan. Previously we have shown that *Bt-*Δ*BT1754* exhibits a significant growth defect when grown in MM-fructose, which is consistent with the requirement of *BT1754* for fructose sensing and utilization (Sonnenburg *et al*., 2010). Quantitative RT-PCR analysis of *Bt-*Δ*BT1754* grown in MM-fructose + arabinan (0.25% w/v each) revealed that expression of the *BT0366*-associated PUL increased to levels equivalent to those seen when *Bt* is grown in arabinan alone (Fig. 4A). The loss of *BT1754* and increased *BT0366*-associated PUL expression was coincident with rapid depletion of arabinose but not fructose in MM-fructose + arabinose, demonstrating the loss of the prioritized consumption of fructose over arabinose exhibited by *Bt* (Fig. 4B, right panel). Together these data demonstrate that *BT1754*-dependent fructose utilization is associated with repression of the *BT0366*-associated PUL.

**Fig. 5:**
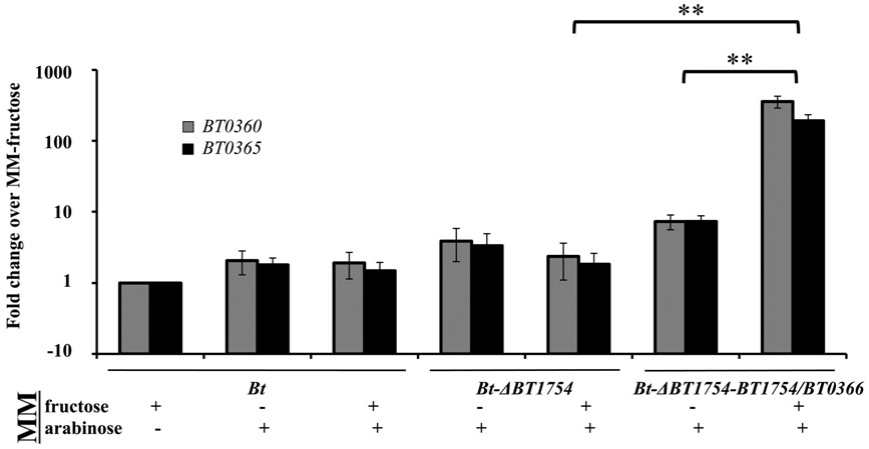
Fructose-dependent upregulation of the *BT0366*-associated PUL requires the presence of the chimeric HTCS *BT1754/0366* and absence of *BT1754*. Relative expression level of *BT0366*-associated genes during *Bt* growth in MM-fructose, MM-arabinose or MM-fructose + arabinose, as determined by qRT-PCR, for *Bt*, *Bt*-Δ*BT1754* and *Bt-*Δ*BT1754–BT1754/BT0366*. Values are mean fold change relative to *Bt* grown in MM-fructose. **P < 0.005. Error bars are ± SE.

We retested the ability of fructose to induce the *BT0366*-associated PUL via the chimeric HTCS, this time expressing *BT1754/BT0366* in the *Bt-*Δ*BT1754* strain, which is unable to impose fructose-dependent repression of the *BT0366*-associated PUL. Due to impaired fructose use of *Bt-*Δ*BT1754*, it is necessary to supply another carbon and energy source to sustain the strain's growth. We reasoned that arabinose would be an ideal sugar to use because it does not induce the expression of the *BT0366*-associated PUL (see Fig. 2A) and as a breakdown product of the inducing arabinose-based ligand, arabinan, it should not have a repressive effect upon this locus. We grew the *Bt-*Δ*BT1754–BT1754/0366* strain in MM-fructose + arabinose, providing conditions in which fructose is not catabolized appreciably but still functions as a ligand for the chimeric HTCS. Assessing expression of the *BT0366*-associated locus by qRT-PCR revealed that the PUL is not upregulated in the background *Bt-*Δ*BT1754* strain lacking the chimera or in the absence of fructose. However, the *BT0366*-associated operon increases expression when the chimeric HTCS and fructose are present and endogenous *BT1754* is absent (Fig. 5). Although our chimera induced expression of the associated PUL, levels were below those induced by endogenous BT0366, indicating that some aspect of our constructed chimera does not perfectly mimic BT0366 activity. Despite the suboptimal upregulation, the data indicate that our chimera functions similarly to the native BT0366 protein. It is important to note that the *BT1754*-associated locus is not induced in the presence of *BT1754/BT0366*, showing that our chimera has the functional specificity of the endogenous BT0366 (Fig. S3). This result confirms that our BT1754/BT0366 chimera functionally reconstitutes PUL regulation that typically depends upon *BT0366* and arabinan in *Bt*. To our knowledge, these data also serve as the first direct demonstration of HTCS signalling independent of substrate catabolism.

### Elucidation of genome-wide regulatory effects of BT1754/BT0366 chimera activation reveals positive and negative regulatory roles of BT0366

**Fig. 6:**
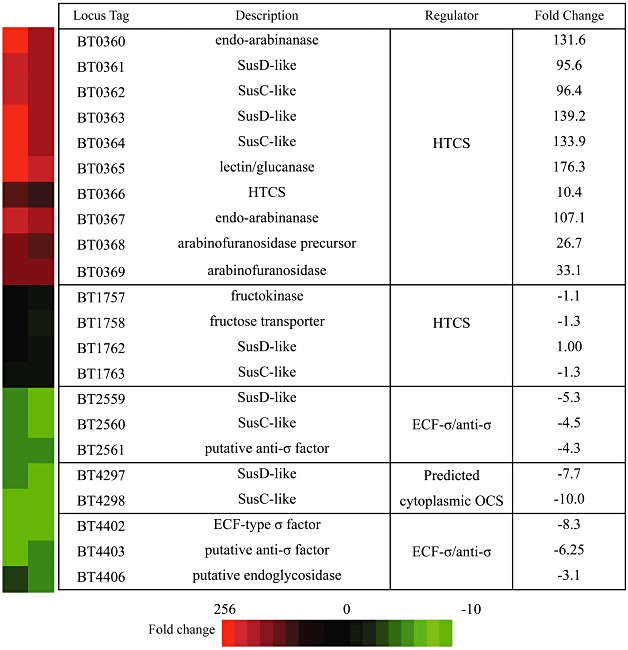
The activated BT1754/BT0366 chimera induces the adjacent PUL and represses distal PULs. Heat map and table displaying genes significantly upregulated or downregulated upon fructose-induced activation of the BT1754/BT0366 chimera. Genes displayed were identified by comparing duplicate GeneChip expression values of *Bt-*Δ*BT1754* and *Bt-*Δ*BT1754–BT1754/BT0366* grown in MM-fructose + arabinose. Genes were only considered for further investigation if more than one gene in a predicted operon was found to be significantly up or downregulated in *Bt-*Δ*BT1754–BT1754/BT0366*. HTCS, hybrid two-component system; OCS, one-component system; ECF-σ factor, extracytoplasmic function σ factor. Fold change represents the mean change of *Bt-*Δ*BT1754–BT1754/BT0366* over *Bt-*Δ*BT1754* for duplicate samples.

The association of many HTCSs with PULs in Bacteroides genomes is consistent with the proximal regulation implied from genetic experiments. HTCS deletion has been shown to result in loss of adjacent PUL expression and the impaired ability of *Bt* to grow on the carbohydrate that induces expression of that PUL. However, it is not clear whether HTCSs can mediate regulation at other sites throughout the genome. To explore this possibility, we next used our functional chimeric HTCS in combination with whole-genome expression profiling to determine the full cohort of genes regulated by BT0366. *Bt-*Δ*BT1754–BT1754/BT0366* was grown to mid-log phase in MM-fructose + arabinose, then profiled using custom *Bt* Affymetrix GeneChips, and the resulting expression profile was compared with *Bt-*Δ*BT1754* (background strain lacking chimera) grown under the same conditions. Analysis of the expression data verified that fructose-induced chimera signalling results in the upregulation of each gene in the *BT0366*-associated operon (average induction across 10 genes = 95.1-fold), but no other operons surveyed were significantly upregulated, demonstrating that BT0366 only significantly induces the adjacent PUL (Fig. 6). Additionally, upon chimera activation several genes within three PULs (*BT2559*–*BT2561*, *BT4293*–*BT4299*, *BT4401*–*BT4407*) exhibited significant downregulation (Fig. 6). These repressed genes are not physically coupled with *BT0366* on the bacterial chromosome, suggesting that BT0366 signalling is also involved in repressing gene expression *in trans*. Interestingly, many recently identified arabinan-responsive genes were not identified in our screen, indicating that regulatory factors other than BT0366 may play a role in a comprehensive response to this polysaccharide (Martens *et al*., 2011).

### BT0366 activation enforces repression of mucus carbohydrate-responsive PULs

**Fig. 7:**
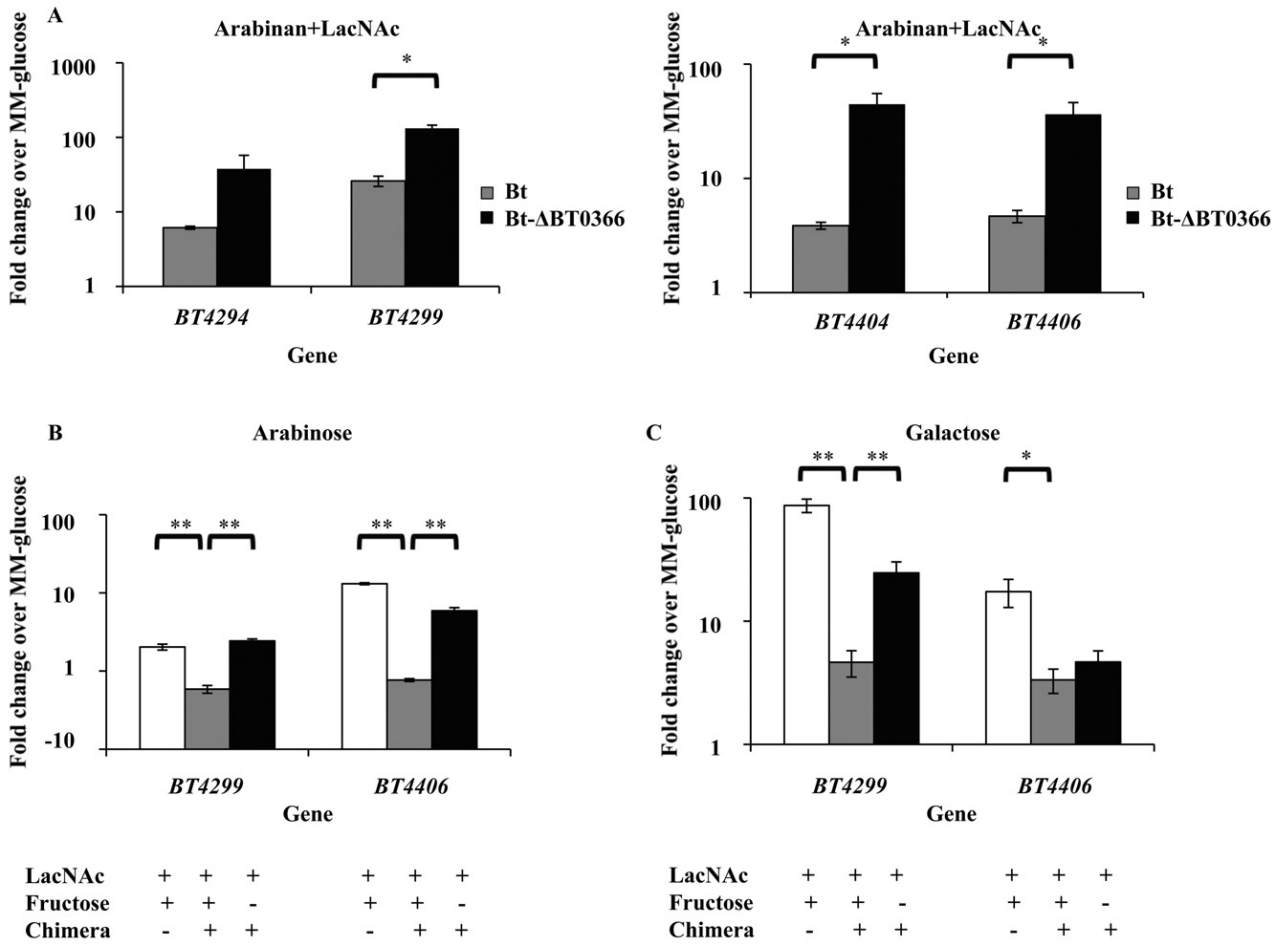
Activated BT0366 represses expression of distal PULs associated with mucus carbohydrate consumption. A. Relative expression levels of the mucus-utilizing, LacNAc-responsive PULs, *BT4294–9* and *BT4404–6*, in *Bt* and *Bt-*Δ*BT0366* grown in MM-arabinan + LacNAc. B. Relative expression levels of *BT4299* and *BT4406* in *Bt-*Δ*BT1754* with or without the *BT1754/BT0366* chimera grown in MM-LacNAc or MM-LacNAc + fructose. Arabinose is added to the media to facilitate growth of *Bt*. C. Same as (B), but using galactose, a breakdown product of LacNAc, to support growth in place of arabinose. For (A)–(C), values shown are mean fold changes relative to *Bt* grown in MM-glucose. Error bars are ± SE. *P < 0.05, **P < 0.005.

Although the downregulation of the three repressed PULs was modest, we reasoned that in the absence of an appropriate inductive signal for these PULs, basal expression levels would likely already be low, and thus these genes would not be dramatically repressed under these growth conditions. Therefore, we next tested whether repression was more apparent if expression of these loci was first induced. *N*-acetyllactosamine (LacNAc; Galβ1–4GlcNac), a disaccharide that is present in mucus glycans, has been previously shown to support *Bt* growth and leads to the upregulation of both *BT4293–BT4299* and *BT4401–BT4407* (Martens *et al*., 2008). We decided to focus on these two PULs due to the availability of an inducing carbohydrate and the fact that they contained the genes with the largest decreases in expression in our GeneChip experiments. When *Bt* was grown in MM-arabinan + LacNAc, both of the LacNAc-inducible operons were upregulated in comparison with expression in glucose (*BT4294*: 6.1-fold induction, *BT4299*: 26.0-fold induction; *BT4404*: 3.9-fold induction, *BT4406*: 4.7-fold induction) (Fig. 7A). However, after deleting the endogenous copy of *BT0366*, these operons significantly increased expression (fold induction over glucose, *P*-value of repression: *BT4294*: 37.8-fold, *P* = 0.16, *BT4299*: 132.2-fold, *P* = 0.0003; *BT4404*: 44.6-fold, *P* = 0.009, *BT4406*: 36.4-fold, *P* = 0.02). These data are consistent with repression mediated by BT0366 signalling being lost in the *Bt-*Δ*BT0366* mutant strain.

We next directly tested whether BT0366 signalling alone can mediate repression of the mucin glycan-responsive PULs. Using fructose-induced signalling of the chimeric BT1754/BT0366 HTCS, we assessed whether repression of the PULs *BT4293–BT4299* and *BT4401–BT4407* would occur upon BT0366 activation without the associated metabolic processing of arabinan. PUL expression was assessed in *Bt-*Δ*BT1754–BT1754/BT0366* grown in minimal medium containing LacNAc, which induces the two mucin-use PULs, with or without fructose, which serves as the activating ligand for the BT1754/BT0366 chimera. Since fructose is not catabolized by this strain but was expected to repress LacNAc via chimera signalling, the conditions required that we also include arabinose in the medium as a carbon and energy source for the cells. Expression of *BT4293–BT4299* and *BT4401–4407* is induced when *Bt-*Δ*BT1754–BT1754/BT0366* is grown in MM-LacNAc + arabinose (Fig. 7B). However, the addition of the chimera ligand fructose to this same medium prevents the full induction of the LacNAc-responsive operons (Fig. 7B; fold decrease for *BT4299* = 4.1-fold, *P* < 10^−4^; for *BT4406* = 7.7-fold, *P* = 0.0001). This repression does not occur when the *Bt-*Δ*BT1754* strain lacking the chimera is grown in identical conditions, illustrating that the fructose-mediated repression depends upon the presence of the chimeric HTCS (Fig. 7B). Together, these data demonstrate that activation of BT0366 is not only responsible for inducing expression of the adjacent PUL, but also leads to repression of distal PULs.

We noticed that the unrepressed expression levels of the LacNAc-responsive genes were lower during growth facilitated by the monosaccharide arabinose than those facilitated by arabinan. These data indicate that *Bt*'s use of arabinose may be leading to an alternative mode of repression (e.g. catabolite repression) of the LacNAc-responsive loci. To test if arabinose was the cause of low expression levels, we replaced arabinose with the monosaccharide galactose, one of the monosaccharide components of LacNAc, which is unlikely to lead to catabolic repression of these LacNAc-responsive loci. We again found that the fructose-activated chimeric HTCS was able to repress the LacNAc-responsive loci in a manner similar to that in the previous experiment, while the expression levels of these loci were higher than in the arabinose-aided growths (Fig. 7C; fold decrease upon fructose addition: *BT4299* = 5.3-fold; *P* = 0.004; *BT4406* = 1.4-fold; *P* = 0.33). These results further support HTCS-mediated repression and also indicate that multiple mechanisms govern efficient expression of the 88 PULs and the resulting carbohydrate harvest and utilization in *Bt*.

### Activated BT0366 directly binds DNA at the PULs it regulates

We performed Chromatin ImmunoPrecipitation with high-throughput sequencing (ChIP-seq) to determine the genomic binding sites of BT1754 and BT0366 upon activation. *Bt* and *Bt-*Δ*BT1754–BT1754/BT0366* were grown in MM-fructose + arabinose, then protein–DNA associations were cross-linked. Following DNA fragmentation, both samples were subjected to immunoprecipitation using antibodies that recognize the periplasmic sensing domain of BT1754. After reversing the cross-links, the fragmented DNA was sequenced using high-throughput Illumina sequencing. Immunoprecipitated sequence sets from *Bt-*Δ*BT1754–BT1754/BT0366* and *Bt* were then compared to determine regions that were bound by the regulatory domains of BT0366 and BT1754, respectively. By growing and processing both strains in the same conditions using the same antibody, we reduced the chances of falsely identifying HTCS-associated loci due to factors such as unrelated changes in bacterial metabolism, differences in antibody affinity, and immunoprecipitation efficiency.

**Fig. 8:**
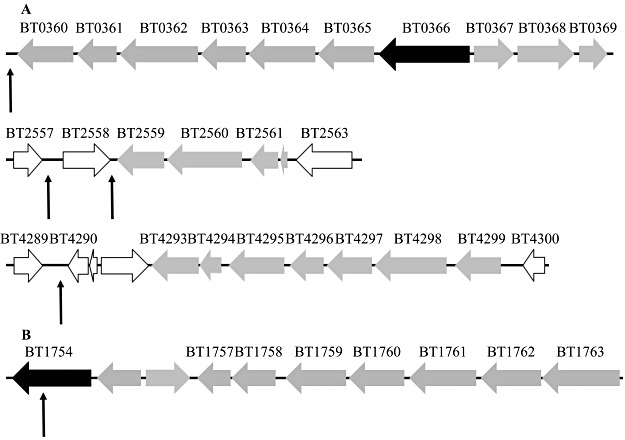
Regulatory domains of BT0366 directly bind to both positive and negative regulatory targets. A. Selected genomic binding targets of activated BT0366 as determined by ChIP-seq using antibodies against the periplasmic domain of BT1754 in the *Bt-*Δ*BT1754–BT1754/BT0366* strain (for a full listing of loci identified see Table S1). Enriched targets were determined via comparison with identically immunoprecipitated samples from the *Bt* strain. B. Selected genomic binding targets of activated BT1754 as determined by ChIP-seq using antibodies against the periplasmic domain of BT1754 in the *Bt* strain (for a full listing of loci identified see Table S2). Enriched targets were determined via comparison with identically immunoprecipitated samples from the *Bt-*Δ*BT1754–BT1754/BT0366* strain. Arrows indicate approximate regions identified by ChIP-seq. For exact genomic co-ordinates, see Tables S1 and S2. Black genes: HTCS; grey genes: PUL-associated genes.

ChIP-seq of the chimeric BT1754/BT0366 HTCS identified a binding region at the BT0366-associated PUL, demonstrating that this HTCS directly binds the DNA adjacent to genes that it upregulates upon activation (Fig. 8A). Additionally, the chimeric HTCS co-precipitated with genomic regions near several PULs found to be downregulated during BT0366 activation including BT2559–2561 and BT4293–4299 (see Fig. 6). These data demonstrate that BT0366 not only directly binds to loci that it positively regulates, but also directly associates with loci that it represses (Fig. 8A, see Table S1 for an annotated list of DNA-binding regions). The most significantly enriched genomic region found upon immunoprecipitation of *B. thetaiotaomicron*'s endogenous BT1754 was the 3′ region of the *BT1754* gene, consistent with this protein impacting regulation of its associated PUL through direct genomic binding (Fig. 8B, see Table S2 for an annotated list of DNA-binding regions).

In addition to the confirmation that these HTCS proteins bind DNA adjacent to the genes that they regulate, this technique allowed us to answer an additional fundamental question about HTCS activity: do intact membrane spanning HTCS directly bind genomic DNA upon activation? By using antibodies directed to the periplasmic domain of BT1754, we were able to retrieve DNA sequences from the loci that these HTCSs are known to regulate. These data confirm that intact (i.e. non-proteolysed) HTCSs are able to directly bind DNA while presumably tethered to the inner membrane. How the activated HTCSs efficiently find their respective DNA targets despite membrane localization and the details of how the DNA regions that they bind influence gene regulation are important topics for future studies. To our knowledge, this study serves as the first demonstration that an HTCS simultaneously regulates multiple sites in the genome, enabling prioritization of carbohydrate substrates by a gut resident Bacteroides species.

## Discussion

One of the defining features of the Bacteroides is the broad diversity of polysaccharides that each species is capable of processing (Xu *et al*., 2007; Martens *et al*., 2008). However, in the dynamic environment of the intestine where multiple carbohydrate substrates of varying relative value may be present at the same time, it is not clear how a cell endowed with > 80 PULs is able to co-ordinate inductive and repressive events into a rapid and optimized transcriptional response. *Bt*'s prioritized utilization of carbohydrates in rich medium has previously been demonstrated (Sonnenburg *et al*., 2006). While HTCSs are known to mediate upregulation of PULs, the mechanisms that underlie the repression of loci for which inductive substrates are available (i.e. the mechanism of prioritization) represent a large gap in our understanding of carbohydrate use in the intestinal ecosystem. Previously, it has been difficult to uncouple the sensor-based induction of an operon from the presence of the native inductive signal. Therefore, the cascades of diverse molecular events that ensue when a PUL's gene products act upon the associated carbohydrate have been difficult to disentangle from the signalling events mediated by the sensors of that same carbohydrate.

Our chimeric HTCS has allowed us to probe the regulatory network of the HTCS BT0366. Whole-genome expression profiling confirmed that BT0366 plays a localized inductive role, confined to upregulating the adjacent PUL. This approach also revealed that BT0366 aids in enforcing a hierarchy of carbohydrate utilization by repressing multiple PULs *in trans*, which would have been difficult to reveal using other methods. In addition, ChIP-Seq experiments revealed that BT0366 associates with its positive and negative regulatory targets in a direct manner. The extent to which other molecules play a role in this process is beyond the scope of this study, but we speculate that other factors likely assist in determining whether BT0366 functions in an inductive or repressive fashion. This repressive regulatory role is novel for the HTCS family, and is likely to be extended to other members of this class of sensors. The development of the chimeric HTCS was key to making these observations and can be extended to explore other members of the HTCS family that lack known ligands. Employing well-defined ligand-binding domains with the chimera HTCS strategy may help define the genomic targets of other members of this class of sensors, not only in *Bt,* but also in the numerous other species that contain members of the HTCS family.

While much remains to be determined about the mechanisms of signal transduction and the biological roles of HTCSs, a hypothesis emerges from our data of why HTCSs are preferred over canonical two-component systems for regulating PULs. The histidine kinases of two-component systems are bifunctional, phosphorylating the response regulator when actively signalling, and de-phosphorylating when the ligand is no longer present. The adjacency of the response regulator to the histidine kinase within the single polypeptide of an HTCS may enable rapid de-phosphorylation upon signalling cessation, thus quickly alleviating repression of other PULs when the higher priority substrate has been depleted. Therefore, HTCSs may provide a kinetic advantage over canonical two-component systems when stepping between the different rungs of gene expression in the carbohydrate consumption hierarchy. How the membrane bound DNA-binding domain of an HTCS overcomes the challenge of finding and binding appropriate regions of the chromosome remains to be determined.

While HTCSs appear to play a role in the establishment of a carbohydrate-consumption hierarchy, the extent of hierarchies within a strain and how many additional mechanisms contribute to their enforcement is still unclear. The inability to prioritize highly valuable carbohydrates over others may cause less valued substrates to act as molecular ‘distractions’ that would likely lead to significant negative selection in an environment as competitive as the mammalian intestine. Efficient prioritization may help to explain the intestinal success of the Bacteroidetes, many members of which possess expanded repertoires of HTCSs.

The distribution of HTCSs across bacterial phyla and whether they maintain conserved functionality in distinct microbial lineages are additional important issues. Interestingly, HTCSs have been found in the sequenced genomes of several bacteria that are neither members of the Bacteroidetes nor residents of the intestinal microbiota. For example, the soil-dwelling δ-proteobacteria *Sorangium cellulosum* contains several annotated hybrid two-component sensor/response regulators (Schneiker *et al*., 2007). Despite the differences in habitat and evolutionary history between *Bt* and *S. cellulosum*, both have devoted large portions of their genomes to a variety of environmental sensors and both have vast carbohydrate consumption capabilities that include plant polysaccharides. These similarities may provide insight into the environmental pressures that promote the evolution and retention of HTCSs.

An in-depth understanding of HTCS-dictated nutrient hierarchies would have significant implications for predicting the effects of diet on the intestinal microbiota and the resulting impact on human health. Dietary supplementation is gaining promise as a means of manipulating the intestinal microbiota to alter host health. Critical to the success of using diet to rationally manipulate the microbiota is understanding how bacteria actually sense and respond to dietary substrates. The expanded presence of HTCSs within the Bacteroidetes suggests that this family of sensor regulators makes key contributions to community responses within the heterogeneous nutritional environment of the gut in which substrates are not all equally valued. Thus, inter-individual variation in HTCS content within a microbiome may explain why specific nutrients have divergent impacts on individuals' respective microbiotas. Focusing on this unique family of proteins may provide an important avenue for more precisely manipulating our resident microbial communities to positively impact human health.

## Experimental procedures

### Reagents, strains and culturing

*Bacteroides thetaiotaomicron* cultures were grown overnight in TYG media then subcultured in minimal media as described previously (Martens *et al*., 2008). Briefly, minimal medium contained 100 mM KH_2_PO_4_ (pH 7.2), 15 mM NaCl, 8.5 mM (NH4)_2_SO_4_, 4 mM l-cysteine, 1.9 mM haematin + 200 mM l-histidine, 100 mM MgCl_2_, 1.4 mM FeSO_4_, 50 mM CaCl_2_, 1 mg ml^−1^ vitamin K_3_ and 5 ng ml^−1^ vitamin B_12_ supplemented with carbohydrate(s) to a final total concentration of 0.5% (w/v) (Martens *et al*., 2008). All reagents were purchased from Fisher Scientific or VWR unless noted otherwise. Glucose, fructose and l-arabinose were purchased from Alfa Aesar (Ward Hill, MA). Sugar beet arabinan (catalogue number P-ARAB) was purchased from Megazyme (Bray, Wicklow, Ireland). *N*-acetyllactosamine (LacNAc) was purchased from V-labs (Covington, LA). Minimal media growth curves were generated by a Powerwave 340 (Biotek, Vinooski, VT) taking readings every 30 min at OD 600 nm. Growths were performed at 37°C in an anaerobic chamber containing a gas mix of 10% CO_2_, 5% H_2_ and 85% N_2_.

### Chimera creation

The *BT1754/BT0366* chimera cloning constructs were created with sewing PCR using the Platinum PFX PCR kit (Invitrogen, Carlsbad, CA) (see Table S4 for primers). The final product consisted of the region (including the 5′ intergenic region plus aa 1–376) of *BT1754* fused to the C-terminal region of *BT0366* (aa 855–1420). The secondary PCR product was cloned into the pNBU2-Erm vector and transfected into *s17-λpir E. coli*, then conjugated into *Bt* using methods previously described (Wang *et al*., 2000; Martens *et al*., 2008). This vector allows for stable genomic insertion adjacent to one of two serine-tRNA sites (Wang *et al*., 2000). Genomic insertions were verified with PCR and mutation-free sequence was confirmed in the final construct via sequencing.

### Gene deletion

All in-frame unmarked deletions in *Bt* were created using a counter selectable allele exchange method as previously described (Koropatkin *et al*., 2008; Martens *et al*., 2008). Briefly, we cloned a construct containing approximately 850 bp of the genomic region directly flanking both sides of the targeted gene plus a start and stop codon into the pExchange vector, which provides an erythromycin-resistance gene and a thymidine kinase (*tdk*, *BT2275*) that complements the *BT2275* deficiency of the parent strain. Subsequent rounds of positive (erythromycin) and negative (5-fluoro-2-deoxyuridine) selection results in an unmarked deletion strain. Deletions were screened with PCR and sequenced to verify frame (see Table S3 for full list of strains used in this manuscript, and see Table S4 for full list of primers used for cloning).

### qRT-PCR transcriptional analysis

*Bacteroides thetaiotaomicron* was grown to mid-log phase in minimal media supplemented with a final total concentration of 0.5% (w/v) of the indicated carbohydrate(s). Bacteria were harvested in RNAProtect (Qiagen, Germantown, MD) following the manufacturer's protocol. The resulting bacterial pellets were stored at −20°C until RNA was extracted with the RNEasy Kit (Qiagen) according to the manufacturer's protocol. cDNA was generated using the SuperScript II Reverse Transcriptase (Invitrogen), and qRT-PCR was performed using cDNA at 10 ng well^−1^, Brilliant SYBR Green (Agilent, Santa Clara, CA) and gene-specific primers as noted in Table S5. Assays were conducted on a Stratagene Mx3000P plate reader. 16S rRNA sequences were used as internal controls and fold changes were calculated using the ΔΔC_T_ method. All values shown are mean values from at least three separate growths each assayed in triplicate.

### *Bt* GeneChip data generation and analysis

RNA from duplicate growths of each condition was collected as described above. Generation of cDNA targets and hybridization to Affymetrix GeneChips was performed as previously described (Martens *et al*., 2008). Briefly, RNA was treated with DNase I to remove contaminating genomic DNA. Superscript II Reverse Transcriptase was used to generate cDNA, which was then fragmented by DNase I treatment, followed by biotinylation. Biotinylated cDNA was hybridized to GeneChips and visualized using laser confocal fluorescent scanning at the Stanford Protein and Nucleic Acid Facility (http://pan.stanford.edu). Data were normalized by RMA normalization in R (http://www.R-project.org/) and comparisons were performed using Significance Analysis of Microarrays (SAM) (Tusher *et al*., 2001). Statistical parameters used for screening the gene list were *q*-value < 1% and fold change > 3-fold. Only predicted operons containing at least two genes meeting the statistical thresholds were considered for this study.

### HPAEC-PAD

HPAEC-PAD was performed at the Glycotechnology Core Resource at the University of California-San Diego (La Jolla, CA). Briefly, cultures were grown in minimal media supplemented with 0.25% (w/v) of each fructose and arabinose. Bacteria were pelleted by centrifugation at indicated times post inoculation and resulting supernatants were acid-treated, passed through a SepPak C18 cartridge, then analysed using a CarboPac column.

### ChIP-seq

Chromatin immunoprecipitation protocols were based on previously described procedures (Aparicio *et al*., 2004; Markel *et al*., 2011) Briefly, *Bt* and *Bt-*Δ*BT1754–BT1754/BT0366* were grown to mid-log phase in 200 ml of MM-fructose + arabinose. Protein–DNA complexes were cross-linked by incubating at room temperature in 1% formaldehyde. Excess formaldehyde was quenched with 0.32 M glycine for 5 min at room temperature, after which cells were pelleted at 4°C and washed two times in ice-cold TBS. Pellets were stored at −20°C overnight, then were thawed and incubated for 10 min at 37°C in 1 ml of CelLytic B Lysis reagent (Sigma-Aldrich, St. Louis, MO), 10 µl of Longlife Lysozyme (G Biosciences, Maryland Heights, MO) and 1 mM PMSF (Sigma-Aldrich). Samples were then sonicated on ice 20 times for 10 s each with a Bronson Digital Sonicator at 45% amplitude, producing fragments of predominantly 100–600 bp. Insoluble material was pelleted at 4°C, then complexes were immunoprecipitated with Dynabeads-Protein G (Invitrogen) bound with 330 µg of polyclonal anti-BT1754 rabbit IgG (a kind gift from David Bolam) according to manufacturer protocol, and eluted in 20 µl of Elution Buffer. Cross-link reversal and protein degradation was performed by adding 20 µl of Pronase (Roche, Indianapolis, IN) in 60 µl of TBS then incubating at 42°C for 2 h and 65°C for 6 h. A Qiagen PCR Purification kit was then used to purify DNA. Library construction was conducted using fragments in the 250–400 bp range and Illumina HiSeq2000 50 bp sequencing was performed at the Duke Genome Sequencing and Analysis Core (Durham, NC). Sequencing reads were analysed with Genomics Workbench (CLC Bio, Cambridge, MA) using default settings with 250 bp read shifts.
