# Supplementary material for: Prioritization of a plant polysaccharide over a mucus carbohydrate is enforced by a Bacteroides hybrid two-component system
Source: Mol Microbiol. 2012 Jul 5;85(3):478–91. doi: 10.1111/j.1365-2958.2012.08123.x (PMC3404733; doi:10.1111/j.1365-2958.2012.08123.x)
Supplement: Supplementary file 1 [file mmi0085-0478-SD1.pdf]

Supporting Information

Prioritization of a Plant Polysaccharide over a Mucus Carbohydrate is Enforced by a Bacteroides Hybrid Two-Component System

Jonathan B. Lynch, and Justin L. Sonnenburg

Department of Microbiology and Immunology, Stanford University School of Medicine
Stanford, California, 94305

For correspondence:
Email. jsonnenburg@stanford.edu
Tel. 650-721-1510
Fax. 650-498-7147

Fig. S1. Growth curves of *Bt*, *Bt-ΔBT1754*, and *Bt-ΔBT0366* strains in various carbohydrates.
Bt, *Bt-ΔBT1754*, and *Bt-ΔBT0366* strains were grown in MM supplemented with 0.5% final concentration of the noted carbohydrates. Absorbance measurements were taken at 600nm (OD_{600}). Values shown are mean of triplicates of each condition.

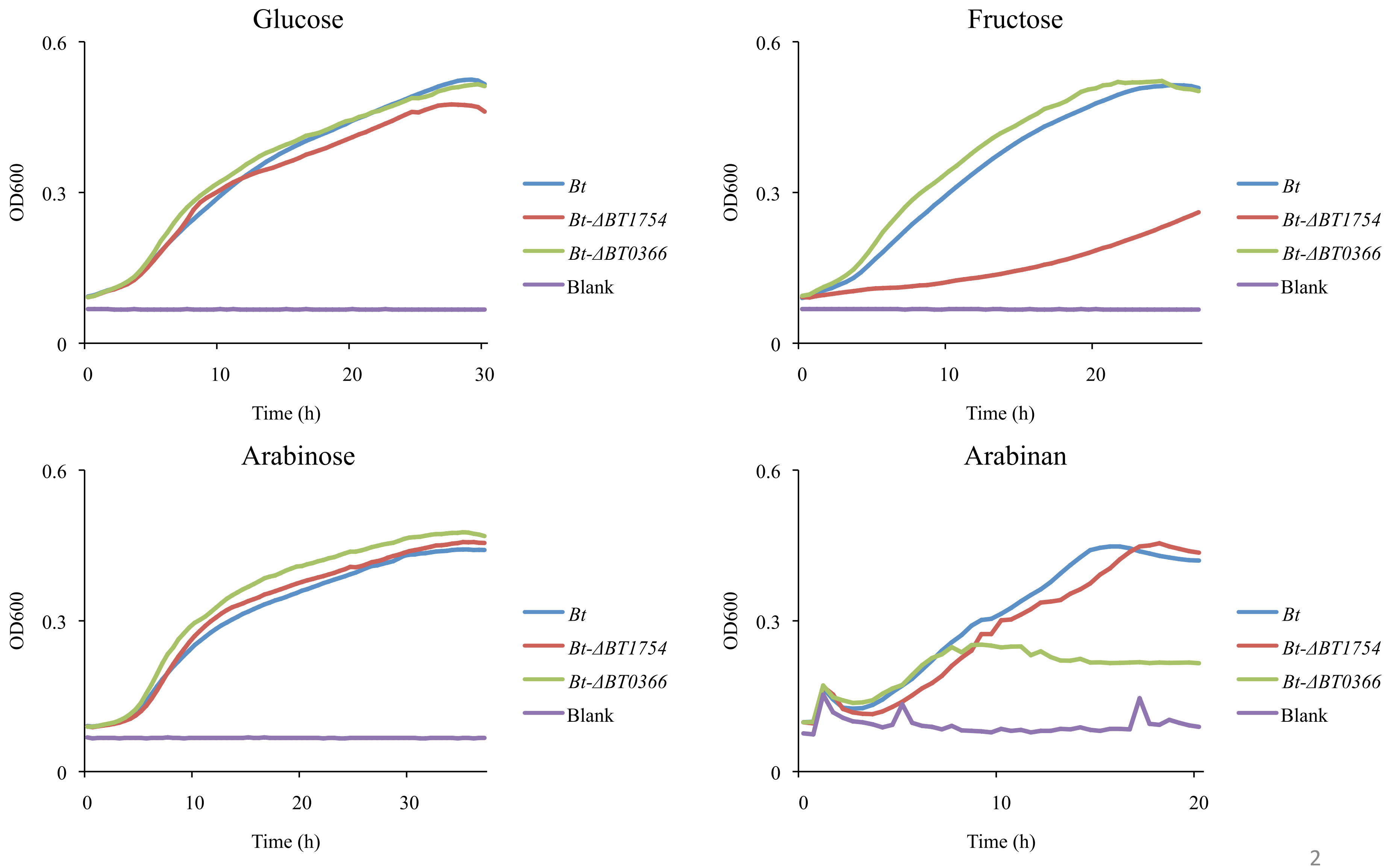

Fig. S2. Deletion of *BT1754* prevents up regulation of the *BT1754*-associated operon during growth in fructose. *Bt* and *Bt-ΔBT1754* were grown in MM supplemented with fructose, arabinan, or both, and expression of the *BT1754*-associated fructokinase *BT1757* was assessed by qRT-PCR. Values shown are mean fold change over *Bt* grown in MM-arabinan. Error bars are +/- SE.

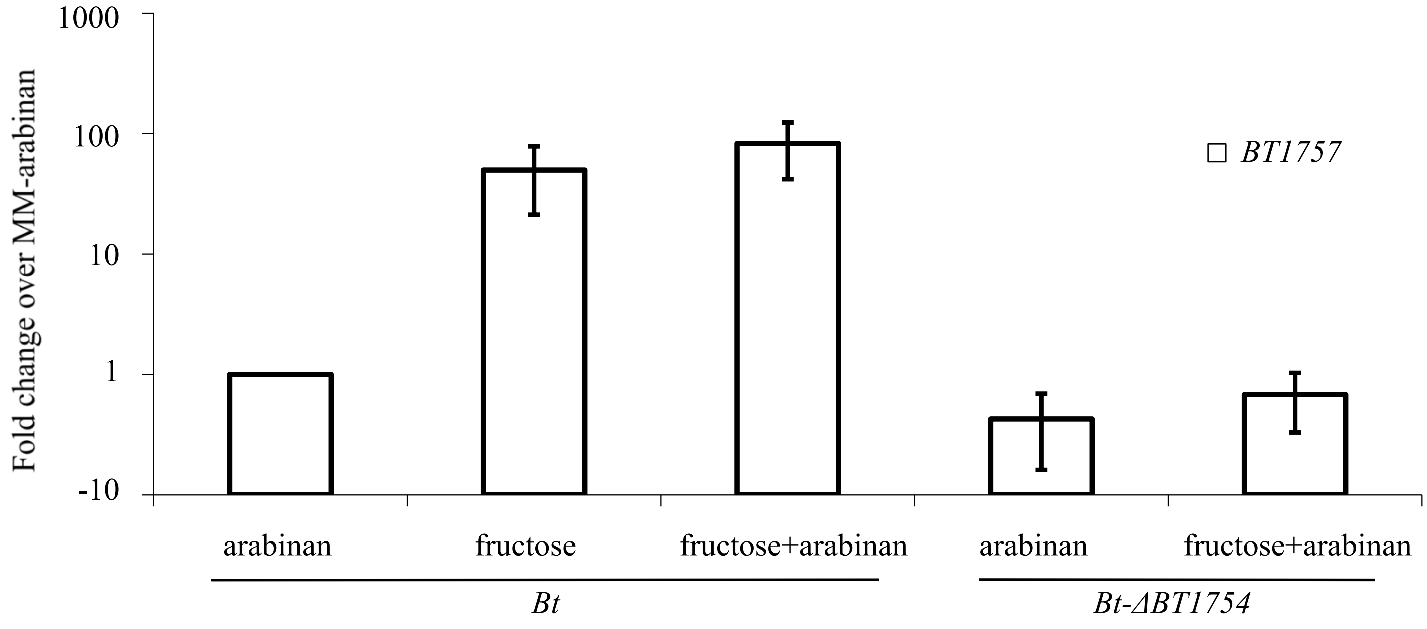

Fig. S3. The *BT1754/BT0366* chimera does not affect *BT1754*-associated genes.
Relative expression of the *BT1754*-associated PUL gene, *BT1757*, in *Bt*, *Bt-ΔBT1754*, and *Bt-ΔBT1754-BT1754/BT0366* grown in MM supplemented with fructose, arabinose, or both; assessed by qRT-PCR. Values represent mean fold change in expression over *Bt* grown in MM-arabinose. Error bars are +/- SE.

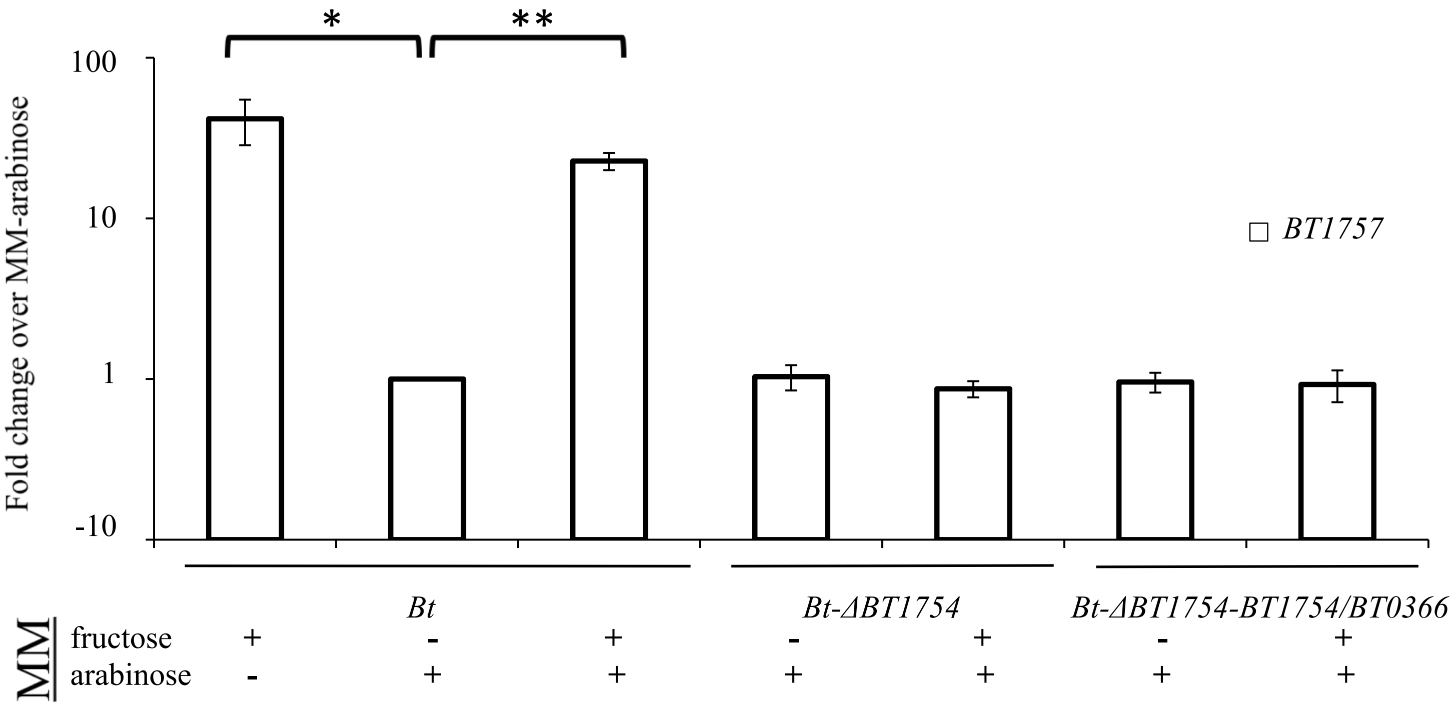

Table S1. List of BT0366 binding loci. Chromatin Immunoprecipitation followed by high throughput sequencing (ChIP-seq) was used to enrich for BT0366 binding loci using an antibody against the periplasmic domain of BT1754 in the *Bt-ΔBT1754-BT1754/BT0366* strain. Enrichment was determined via comparison with sequences derived from the same immunoprecipitation performed in the *Bt* strain. Datasets were analyzed with CLC Genomics Workbench using the ChIP-seq toolkit.

Genomic coordinates	False discovery %	Gene name
431842-431855	1.7e-3	BT0359-BT0360 intergenic region
1973275-1973282	1.35e-13	BT1602-BT1603 intergenic region
2336929-2336949	1.58e-4	BT1862-5S intergenic region
2345070-2345092	4.71e-18	BT1866-BT1867 intergenic region
2993445-2993459	0.03	BT2397-BT2398 intergenic region
3190619-3190676	1.6e-15	BT2557-BT2558 intergenic region
3192445-3192449	2.56e-9	BT2558-BT2559 intergenic region
4399596-4399610	6.37e-4	BT3415
4484457-4484472	1.6e-10	BT3478-BT3479 intergenic region
4709598-4709605	4.01e-10	BT3633-BT3634 intergenic region
4816848-4816869	6.86e-6	SusR-BT3706 intergenic region
4930604-4930612	8.06e-13	BT3794-BT3795 intergenic region
4976427-4976442	3.77e-21	BT3824-BT3825 intergenic region
5439702-5439710	1.89e-8	BT4143-BT4144 intergenic region
5650981-5650988	2.43e-5	BT4289-BT4290 intergenic region
5784589-5784597	4.89e-10	BT4391-BT4392 intergenic region
5999278-5999322	5.73e-6	BT4579-BT4580 intergenic region
6220119-6220127	4.82e-14	BT4740-BT4741 intergenic region

Table S2. List of BT1754 binding loci. Chromatin Immunoprecipitation followed by high throughput sequencing (ChIP-seq) was used to enrich for BT1754 binding loci using an antibody against the periplasmic domain of BT1754 in the *Bt* strain. Enrichment was determined via comparison with sequences derived from the same immunoprecipitation performed in the *Bt-ΔBT1754-BT1754/BT0366* strain. Datasets were analyzed with CLC Genomics Workbench using the ChIP-seq toolkit.

Genomic coordinates	False discovery %	Gene name
2165495-2165595	0.00	BT1754
4399582-4399606	2.68e-24	BT3415
5439720-5439726	4.3e-8	BT4143-BT4144 intergenic region

Table S3. Strains referenced in this paper

Name	Description	Reference
Bt	Parent *Bt* strain VPI-5482 Δ*tdk*	(Koropatkin *et al.*, 2008)
Bt-ΔBT0366	Parent w/ in frame deletion of HTCS *BT0366*	This paper
Bt-ΔBT1754	Parent w/ in-frame deletion of HTCS *BT1754*	(Sonnenburg *et al.*, 2010)
Bt-BT1754/BT0366	Parent containing *BT1754/BT0366* chimera integrated next to genomic Ser-tRNA$_{UGA}$ site	This paper
Bt-ΔBT1754-BT1754/BT0366	*Bt-ΔBT1754* strain with *BT1754/BT0366* chimera integrated at genomic Ser-tRNA$_{UGA}$ site	This paper

Table S4. Cloning primers used in this paper

Primer name	Sequence
BT1754 forward primary	GGAAACGCTACACTATTGATGGTAAA
BT1754/BT0366 sewing reverse	TTCTAACAGATGCTTTTGCTGTTCAAGCTCTTTATTCAAGCGA TTCTTAG
BT0366 reverse primary	CCCAATCCTGACAAGAACTCAT
BT1754/BT0366 sewing forward	CTAAGAATCGCTTGAATAAAGAGCTTGAACAGCAAAAGCAT CTGTTAGAA
BT1754 forward secondary	GAAAAGGAATGGGATCCAAACTAA
BT0366 reverse secondary	GCTGAGAACGTCTAGAATTGCCAT
0366FragAfwrd	CTGTTTGATCTGTTCCGGAGAC
0366sewR	GCCCTTATTAATACGTACATATATATGTAACTGATATAAGGG TTGCTATTATGAAAC
0366sewF	GTTTCATAATAGCAACCCTTATATCAGTTACATATATGCACG TATTAATAAGGGGC
0366FragBrev	TTATTTGATTCTTTTTCCCCAGAC
0366F. secondary	CCCGGATCGGGATCCCCCCAGTCG
0366R.secondary	GTTTCCTTCACCGTCTAGAAAAGACGGC

Table S5. qRT-PCR primers used in this paper

BT16S-F4	GTGTAGCGGTGAAATGCTTAGATATC
BT16S-R4	CAGTGTCAGTTGCAGTCCAGTGA
BT1757-1F	ATGGGATAACATTCCTTTCACG
BT1757-1R	AGACTCGCGCAATACTTCTTTC
BT1763-1F	AGTAGGCAAACATCGTGGAGAT
BT1763-1R	GAGCGAGTGAATACCCCTCAC
BT0360.1F	GTCAAACGGGGATTCTGCTA
BT0360.1R	GTTGGCAAGGTTCCAGTCAT
BT0365.1F	CCGCACAAAATGAGAAAGGT
BT0365.1R	GCCGATACTGTCCATGAGT
BT4294aF	TAGACTCCACCCGAAAATGC
BT4294aR	TGCATATCTTTGGCAGCTTG
BT4299aF	GGCAAAGCAGGAAGTGACTC
BT4299aR	AGCCCTTCCCGTTCATAACT
BT4404aF	CTATTCTGAGCGGACCTTCG
BT4404aR	AGTTGGTTCCGGTGTTGAAG
BT4406aF	GCGGTAAGATCACGGTTTGT
BT4406aR	CTGCGATATGGTCTTCAGCA
